# Design, Construction and Immunogenicity Assessment of pEGFP-N1-KMP11-GP96 (Fusion) as a DNA Vaccine Candidate against *Leishmania major* Infection in BALB/c Mice

**Published:** 2020

**Authors:** Abdolhossein DALIMI, Vahid NASIRI

**Affiliations:** 1. Department of Parasitology and Entomology, Faculty of Medical Sciences, Tarbiat Modares University, Tehran, Iran; 2. Department of Parasitology, Razi Vaccine and Serum Research Institute, Agricultural Research, Education and Extension Organization, Karaj, Iran

**Keywords:** *Leishmania major*, Fusion gene, Vaccine

## Abstract

**Background:**

KMP-11 (Kinetoplastid membrane protein-Π) exists in all species of kinetoplastid family. It is fully conserved and the protein produced by this gene can induce a very high cellular immune response. We aimed to design a suitable construction for a *Leishmania major* DNA vaccine and evaluate the protective efficacy of it as a candidate for DNA vaccine against cutaneous leishmaniasis in BALB/c mice.

**Methods:**

This experimental study was conducted in Tehran City, Iran, between April 20, 2015 and May 30, 2016. KMP-11 gene of *L. major* (MRHO/IR/75/ER, Iranian strain) and NT-*GP96* of Xenopus *GP96* DNA from a pBluescript-*GP96* plasmid were amplified by PCR and the purified PCR products were cloned into the pJET1.2/blunt plasmid vector, then, subcloned into pEGFP-N1 plasmid as an expression vector. Finally, the KMP-11 gene was fused with *GP96* and afterward the combination cloned in pEGFP-N1. All the cloned genes confirmed by enzyme digestions. Then, four groups of mice were immunized with PBS, pEGFP-N1, pEGFP-N1-KMP, and pEGFP-N1-fusion. Four weeks after immunization, all animals were challenged with *L. major* virulent promastigotes.

**Results:**

The constructed fusion potentially showed an ability to elicit Th1 responses that led to cutaneous lesion healing. Interestingly, the group received KMP11-*GP96* –GFP showed the highest ratio of IFN- γ /IL-4 and IgG2a/IgG1 compare to other groups. No side effect was observed after using the fusion in the mice.

**Conclusion:**

The constructed fusion could well stimulate both the cellular and humoral immune systems that led to cutaneous lesion healing in mice.

## Introduction

Leishmaniasis is a neglected tropical disease that causes human infections varying from self-healing cutaneous lesions to mucosal diffuse cutaneous and visceral forms. Antimonial resistance is a major problem that we faced in controlling the disease in Iran. Considering the increasing of clinical drug resistance (1), introducing a new effective antigen for producing an anti-leishmanial vaccine seems crucial for preventing the disease in endemic areas.

Several antigens of *Leishmania major* that generate appropriate immune responses have been cloned and evaluated in vitro and in the *L. major* infected BALB/c mice model. These antigens were such as gp63 (2), Hsp70 (3), TSA (4), PSA-2 (5), *Histone H1* (6), *LACK* (7, 8), *LACK* and *TSA* (9), *LeIF* (10), *LeIF* and *TSA* (11), *LeIF*, *LACK* and *TSA* (12), *LACK*-*TSA* fusion (13).

Kinetoplastid Membrane Protein 11 (KMP-11) is a complex protein strongly associated with lipophosphoglycan of *Leishmania* promastigotes (14). The lipophosphoglycan is highly antigenic for human and murine T cells (15). This is due to the presence of 11-kDa protein in the protozoan membrane. Among various *Leishmania* molecules known as potential candidate antigens for second-generation vaccines, KMP-11 has concerned much attention because of highly antigenicity for murine, canine and human T cells (16).

*GP96* is a member of the HSP90 family and plays an important role in innate and adaptive immune responses, protein folding and assembly (17). *GP96* can cause pro-inflammatory cytokine secretion, such as IL-12 and GM-CSF, and maturation of APCs (18). Previously, the *GP96* N-terminal domain has a potent adjuvant activity toward the surface antigen of hepatitis B (19). Protective DNA vaccination has been performed using proteins fusion to the *GP96* peptide against *Listeria monocytogenes* in the mice.

The main aim of the present study was to design the pEGFP-N1- KMP 11-NT*GP96* Fusion construction to use it as a *L. major* DNA vaccine.

## Materials and Methods

### Ethics Statement

This research was carried out in accordance with the recommendations in the Guide for the Care and Use of Laboratory Animals of the Tarbiat Modares University. All animal experiments, including maintenance, handling and blood collection was approved by the Institutional Animal Care and Research Advisory Committee of Tarbiat Modares University based on the Specific National Ethical Guidelines for Biomedical Research issued by the Research and Technology Deputy of Ministry of Health and Medicinal Education of Iran.

### Design

**Fig. 1:**
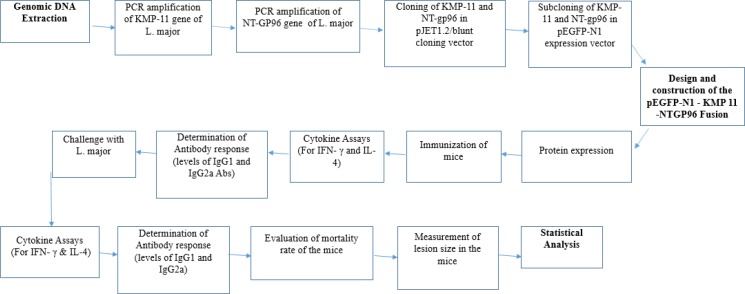
The design, construction and immunogenicity assessment of pEGFP-N1-KMP11-*GP96* (Fusion) in a flow chart

This experimental study conducted in Tehran City, Iran, between April 20, 2015 and May 30, 2016. The design, construction and immunogenicity assessment of pEGFP-N1-KMP11-*GP96* (Fusion) is shown in a flow chart (Fig. 1).

### Parasite and plasmids

The Iranian strain of *L. major* (MRHO/IR/75/ER) was kindly provided by Razi vaccine and serum research institute, Iran. Promastigotes were cultured in RPMI-1640 medium supplemented with 10% FCS (Fetal Calf Serum, Sigma-Aldrich, Germany) and incubated at 24 ± 1 °C. A pBluescript-*GP96* plasmid containing the Xenopus *GP96* DNA (accession number AY187545, 2552 bp) was kindly provided by Dr. Bolhassani (Pasteur Institute of Iran, Tehran, Iran). *Escherichia coli* (*E.coli*) strains TOP10 were received from Novagene Co.,pJET1.2/blunt cloning vector and pEGFP-N1expression vector was obtained from Fermentas and Invitrogen Co., respectively.

### Genomic DNA Extraction

Genomic DNA of *L. major* promastigotes was extracted by commercial DNA Extraction kit (Bioneer, Korea) according to the manufacturer’s protocol. Qualityand purity of extracting DNA were assessed by electrophoresis on the 1% agarose gel and spectrophotometerically.

### Primer design and PCR amplification KMP-11

A pair of oligonucleotide primers were designed based on the KMP-11 gene sequences (accession number KF150697, 279 base pair) according to previous researches (20) as follows:

Forward primer: 5′-AGA TCT ACC ATG GCC ACC ACG TAC GAG GAG-3′ that ACC ATG: Kozak sequence and AGA TCT: *Bgl II* cut site. Reverse primer: 5′-GAA TTC CTT GGA TGG GTA CTG CGC AGC-3′ that GAA TTC: *EcoRI* cut site and primers without stop codon.

The PCR amplification with *Pfu* DNA polymerase (Vivantis) and DNA of *L. major* was done according to: 95 °C for 3 min as initial denaturation, 35 cycles at 95 °C for 30 sec, 60 °C for 30 sec, 72 °C for 30 sec and then 72 °C for 10 min as final extension.

### NT-GP96

The forward and reverse primers for amplifying the NT-*GP96* of Xenopus *GP96* DNA (accession number AY187545, 1014 base pair) were designed as follows:

Forward primer: 5′-CGG GAA TTC GAA GAT GAC GTT GAA -3′ that GAA TTC: *EcoRI* cut site.
Reverse primer: 5′-AT GGT ACC TTT GTA GAA GGC TTT GTA-3′ that GGT ACC: *KpnI* cut site.

The following program was used for PCR amplification of NT-*GP96* with using pBluescript-*GP96* plasmid and Pfu DNA polymerase (Vivantis): 95 °C for 5 min as initial denaturation, 30 cycles at 95 °C for 1 min, 62 °C for 2 min and 72 °C for 1.5 min and then 72 °C for 20 min as final extension.

Finally, the PCR products were analyzed by electrophoresis on 1.2% (w/v) agarose gel. The KMP-11and NT-*GP96* PCR products bands were cut under long (312) UV wavelength and were purified from gel by using a gel purification kit (Bioneer, Korea). Correct insertion confirmed by PCR, restriction enzymes digestion and sending to the Gen Fanavaran ® Company (Iran, Tehran) for sequencing.

### Cloning of KMP-11and NT-GP96 inpJET1.2/bluntcloning vector

The purified gene fragment of KMP-11 and NT-*GP96* was ligated into pJET1.2 cloning vector at 22 °C for an hour and then at 4 °C overnight with T4 DNA ligase (Fermentas Co.). The ligation products were transformed into *E. coli TOP10* strain competent cells and dispersed onto LB agar plates containing 100 μg/ml ampicillin. After overnight incubation at 37 °C, colonies on the agar plate that contained recombinant plasmids were detected. For confirmation, PCR amplifications were performed in these colonies using primers specific for KMP-11and NT-*GP96* genes and colonies containing the recombinant plasmid were selected. Recombinant plasmids were extracted by Vivantis plasmid extraction kit and digested by *Bgl II* / *EcoRI* (for KMP-11), *EcoRI* / *KpnI* (for NT-*GP96*) restriction enzymes (Fermentas Co. ^®^) for digestion confirmation.

### Subcloning of KMP-11and NT-GP96 in pEGFP-N1expression vector

pJET- KMP-11, pJET- NT-*GP96* and pEGFP-N1 were digested by *Bgl II* / *EcoRI* (for KMP-11), *EcoRI* / *KpnI* (for NT-*GP96*) restriction enzymes and the purified gene fragment of KMP-11and NT-*GP96* KMP-11 ligated into digested pEGFPN1expression vector by using of T4 DNA ligase enzyme. Recombinant plasmids pEGFP-KMP-11 and pEGFP -NT-*GP96* were transformed into *E. coli* TOP10 strain competent cells and dispersed onto LB agar plates containing 100 μg/ml of Kanamycin at 37 °C for overnight. Afterward, PCR amplifications were performed in colonies on the agar plate using specific primers of KMP-11and NT-*GP96* genes and colonies containing the recombinant plasmid were selected. Then, recombinant plasmids were extracted by Vivantis plasmid extraction kit and digested by *Bgl II* / *EcoRI* (for KMP-11), *EcoRI* / *KpnI* (for NT-*GP96)* restriction enzymes (Fermentas Co.).

### Design the pEGFP-N1 - KMP 11 -NTGP96 Fusion construct

The recombinant plasmids pEGFP-N1 - KMP 11 and pJET-NT-*GP96* were digested by *EcoRI* and *KpnI* restriction enzymes. The products of digestion were analyzed by electrophoresis on 1.2% agarose gel and the digested bands of NT-*GP96* fragment (1014 bp) and pEGFP-N1-KMP 11 were purified by gel purification kit (Vivantis Co.). NT-*GP96* genes ligated into digested pEGFP-N1-KMP 11 by using of T4 DNA ligase enzyme. Recombinant plasmids pEGFP-N1-KMP11-NT*GP96* were transformed into *E.coli* TOP10 strain competent cells and dispersed onto LB agar plates containing 100 μg/ml of Kanamycin at 37 °C for overnight. Then, colonies on the agar plate that contained recombinant plasmids were detected. For confirmation, PCR amplifications were performed in these colonies using primers specific for KMP-11and NT-*GP96* genes and colonies containing the recombinant plasmid were selected. Recombinant plasmids were extracted by Vivantis plasmid extraction kit and digested by *Bgl II* / *EcoRI* (for KMP-11), *EcoRI* / *KpnI* (for NT-*GP96)* and *NheI* / *KpnI* (for KMP 11-NT*GP96* fusion) restriction enzymes (Fermentas Co.®) for digestion confirmation.

### Protein expression, and verification of protein expression in eukaryotic cells

Previously (40), KMP11-NT*GP96* -GFP Fusion were Subclonned in pLEXSY-neo *Leishmania* expression vector, and recobbinant constructs were transfected into *L. tarentolae* promastigotes as eukaryotic cell, that production of recombinant protein of KMP11-NT*GP96* -GFP gene was confirmed by SDS–PAGE and Western Blotting.

### Mice, Immunization Schedules

Female inbred BALB/c mice, 6-week-old were purchased from Razi Vaccine and Serum Research Institute, Karaj, Iran. They were housed in clean cages and fed ad libitum. The immunization experiments were carried out in four groups of mice (n= 15 at each group) and all tests were done in triplicate. The first group received PBS only and Group 2 immunized with pEGFP-N1; group 3 vaccinated with pEGFP-N1-KMP11; group 4 vaccinated with pEGFP-N1-KMP11-*GP96* (FUSION). All groups were immunized with 100μg of the materials three times (0, 21, 42 d) in the quadriceps muscle. Four weeks after last immunization, all animals were challenged with 2×10 ^6^ stationary phase of *Leishmania major* (MRHO/IR/75/ER) virulent promastigotes by intradermal inoculation in the tail. Cutaneous lesion size was measured by digital vernier caliper every day until 30 wk after challenge.

After 30 wk, with full respect to the principles of medical ethics, all live mice initially anesthetized with halothane as an inhalant anesthetics euthanasia, in a chemical fume hood. Then the animals sacrificed, and their liver, bone marrow and spleen were studied for the presence of the *Leishmania* by smear preparation and cultivation in the culture medium.

### Cytokine Assays

To determine the levels of IFN- γ and IL-4, in each group of experiment five mice were sacrificed before and 4 wk after challenge and spleen of them were removed and homogenized in PBS. After erythrocytes lysis using ACK lysis buffer (0.15 M NH4Cl, 10 mM KHCO3 and 0.1 mM Na2-EDTA), splenocytes were washed with PBS and resuspended in RPMI-10% FCS. Cells were then seeded at a density of 3.5×10 ^6^ cells/ml in the presence of *L. tarentolae*-KMP11-NT*GP96* -GFP Freeze/Thawed (25 mg/ml) that were designed and constructed in previous research (20). Concanavalin A (Con A; 5 mg/ml) and medium alone were used as the positive and the negative control respectively. Plates were incubated for 72 h at 37 °C in 5% CO2 humidified atmosphere for IFN- γ and IL-4 measurement. The IFN- γ and IL-4 production in supernatants of splenocytes cultures was measured by ELISA kits (U-CyTech, Netherlands), according to the manufacturer’s instructions. All experiments were run in triplicates.

### Determination of Antibody response

Before challenge and 4 wk after that, all groups of mice were bled retro-orbitally and the levels of IgG1 and IgG2a Abs were evaluated using ELISA method according to the manufacturer’s instruction (Mouse IgG2a&1 detection kit, eBioscience, USA).

### Statistical Analysis

Statistics were performed using SPSS ver. 18 (Chicago, IL, USA) and one way ANOVA (Multiple-comparison Tukey post Hoc test) and Student’s t-test was employed to assess the significance of the differences between the mean values of control and experimental groups. Differences were considered statistically significant when *P*<0.05. Data shown represent the mean values ± standard error of the mean (SEM) of three independent experiments.

## Results

### Fusion construct

**Fig. 2:**
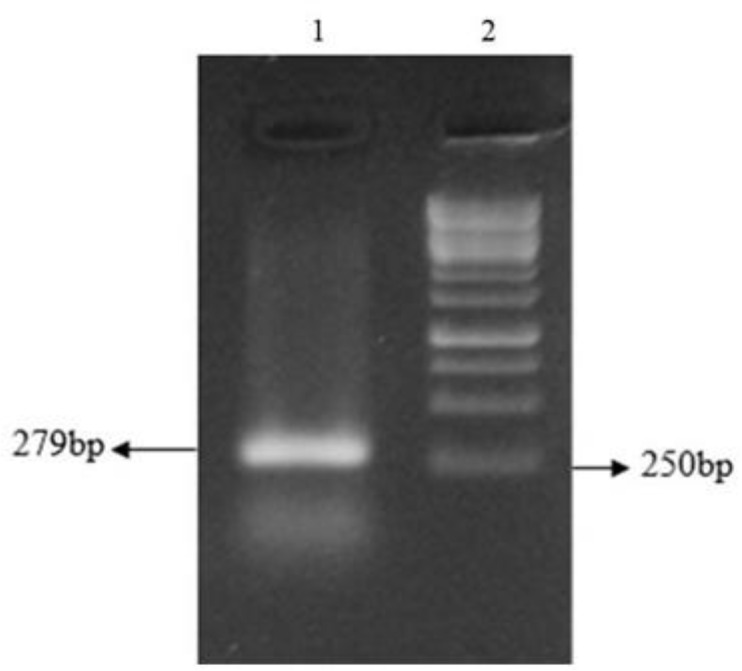
Electrophoresis of the amplified KMP-11 gene of *L.major* on 1.2 % (w/v) agarose gel. Lane 1: single expanded band of KMP-11 gene (approximately 279 bp); Lane 2:100 bp DNA ladder

**Fig. 3:**
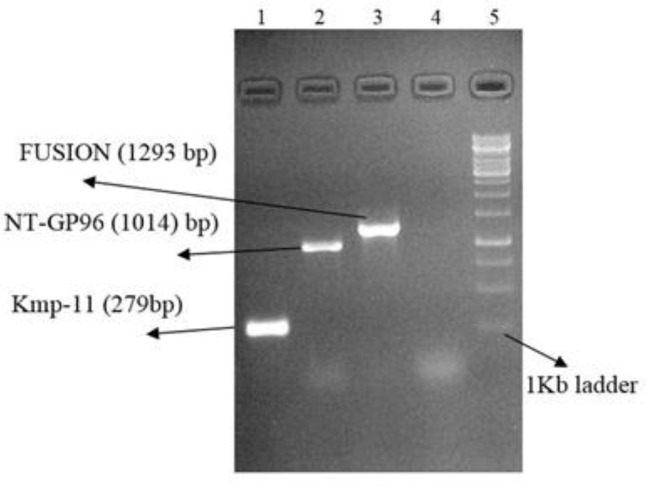
Electrophoresis of the amplified KMP11 of *L.major*, NT-*GP96* and Fusion genes on 1.2 % (w/v) agarose gel. Lane 1: KMP11 gene; Lane 2: single expanded band of NT-*GP96* gene (approximately 1014 bp); Lane 3: Fusion; Lane 4: Negative control; Lane5: 1kb DNA ladder

**Fig. 4:**
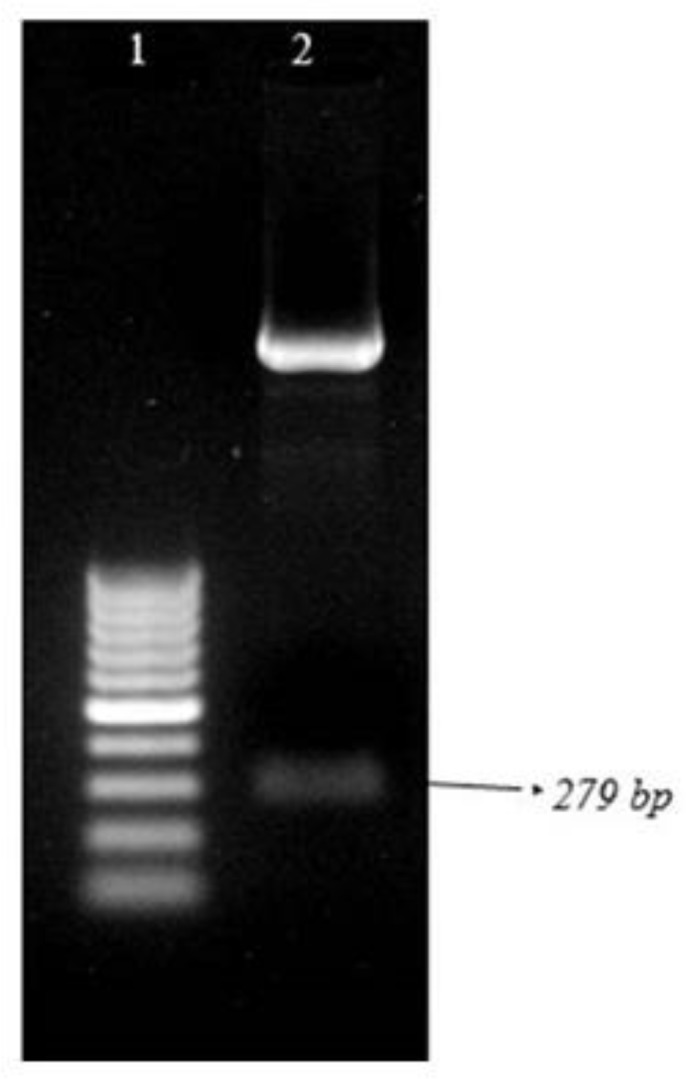
Analysis of enzymatic digestion of pEGFP-N1-KMP11 on 1.2% (w/v) agarose gel. Lane 1: 100 bp Plus DNA ladder. Lane 2: Double digestion of GFP-KMP11 with *Bgl II* and *EcoRI* (279)

Following PCR amplification a 279 bp DNA fragment for KMP-11 and 1014 bp for NT-*GP96* was identified by agarose gel electrophoresis of the PCR products (Figs. 2–4). The PCR products were ligated into a pJET1.2/blunt cloning vector and transformed into *E. coli* TOP10 strain. Bacterial colonies containing the recombinant *pJET*- KMP-11 and pJET-*NT*-*GP96* plasmids were confirmed by PCR using KMP-11, *NT*-*GP96* and *pJET1*.2/blunt specific primers. Subsequently, KMP-11 and *NT*-*GP96* genes were successfully subcloned into pEGFPN1expression vectors and transformed into *E. coli* TOP10 strain. The presence of the inserted genes was confirmed by PCR, using specific primers for them. The extracted pEGFPN1-KMP11 plasmids successfully digested with *Bgl II* and *EcoRI* enzymes that after analyzing on 1.2 %(w/v) agarose gel showed a 279 bp fragment (Fig. 3).

**Fig. 5:**
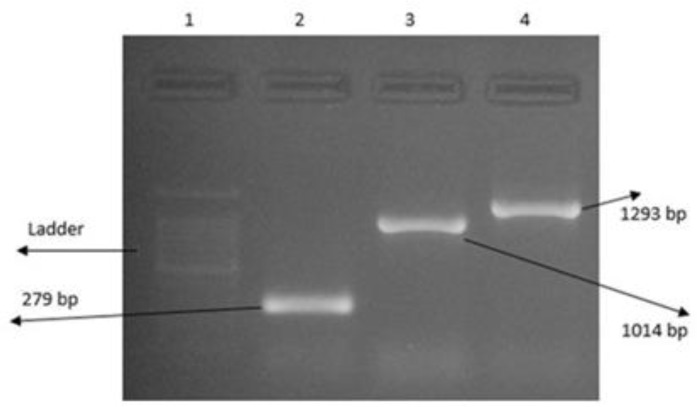
Electrophoresis of the amplified KMP-11, NT-*GP96* and KMP11-NT*GP96* fusion genes in PCR reaction with using recombinant plasmid pEGFP-N1- KMP11-NT*GP96* as the template and genes specific primers that Lane1: 100bp DNA ladder; Lanes 2, 3, 4: expanded bands of KMP-11, NT-*GP96* and KMP11-NT*GP96* fusion genes (approximately 279 bp, 1014 bp and 1293 bp respectively)

*NT-GP96* genes ligated into pEGFP-N1-KMP11 and recombinant plasmid pEGFPN1- KMP11-NT*GP96* was transformed into *E. coli* TOP10 strain. The presence of the inserted construct was confirmed by PCR amplifications using primers specific for KMP-11, *NT*-*GP96* and KMP11-*NTGP96* fusion genes (Fig. 5). Recombinant plasmids successfully digested by *Bgl II* / *EcoRI* (for KMP-11), *EcoRI* / *KpnI* (for *NT*-*GP96)* and *NheI*/*KpnI* (for KMP11-*NTGP96* fusion) restriction enzymes for digestion confirmation.

### The IFN- γ /IL-4 ratio

**Fig. 6:**
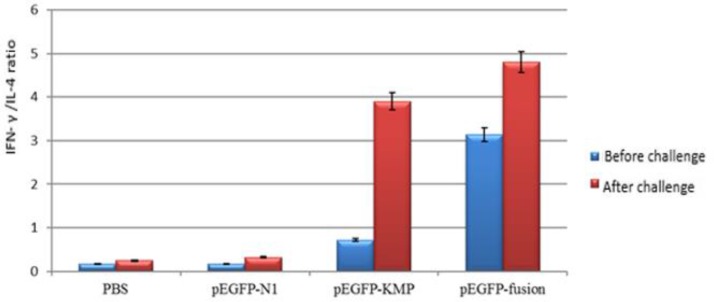
IFN- γ /IL-4 ratio in different groups before and after challenge with *L. major*. The IFN- γ /IL-4 ratio in response to KMP11-NT*GP96* -pEGFP and PBS compared (*P< 0.05)

The levels of IFN-γ and IL-4 production were analyzed before and 4 weeks after challenge in the supernatant of the spleen cells culture of all four groups following stimulation with Freeze/Thawed *L. tarentolae-*KMP11-NT*GP96* -GFP. Stimulation of isolated splenocytes from the vaccinated group with recombinant plasmid pEGFP-N1- KMP11-NT*GP96* prior and 4 wk after challenge elicited a significantly higher IFN-γ production than other groups (*P*<0.05) (Fig. 6).

The calculation of the IFN- γ to IL-4 ratio for each vaccinated group were used as an indicator of potential immunization. The *Leishmania* specific IFN- γ /IL-4 ratio was higher in the pEGFP-N1- KMP11-NT*GP96* vaccinated group compared to the others both at before and 4 wk after challenge.

### IgG antibody isotypes response to Immunization and challenge

**Fig. 7:**
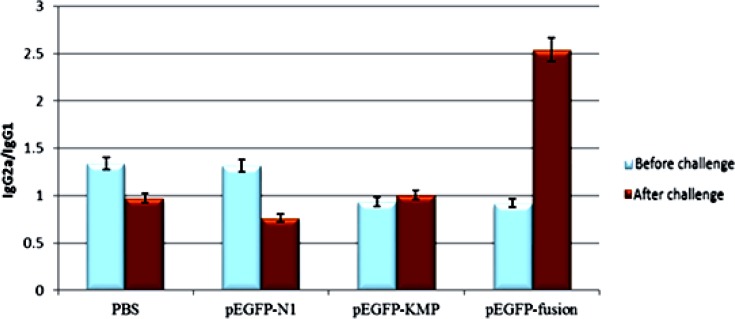
Optical density ratio of serum antibodies (IgG2a/IgG1) from BALB/c before and after challenge with *L. major*. Bars represent the mean+ standard error of the mean (SEM) of optical density values of 15 mice

To compare IgG isotypes in different groups, all sera were assayed by ELISA before and 4 wk after immunization. The ratio of IgG2a/IgG1 was highest in the pEGFP-N1- KMP11-*NTGP96* vaccinated group that the differences were significant as compared with all other groups (*P*<0.05)(Fig. 7) .

### Mortality rate of immunized and control mice after challenge

**Fig. 8:**
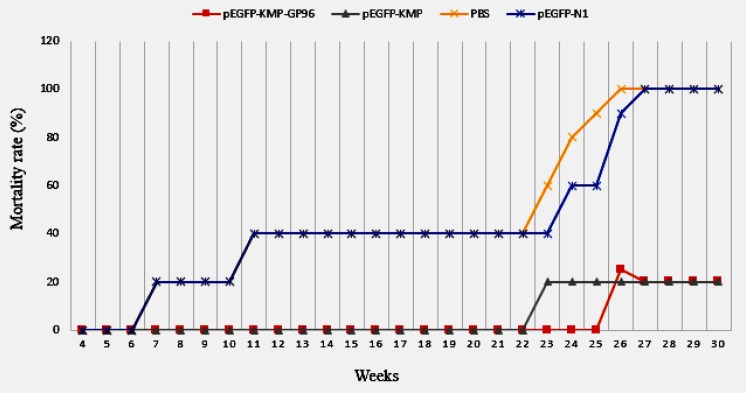
Mortality rates of immunized BALB/c mice after challenge with 2×10 ^6^ promastigotesof L.major 30 weeks after the last immunizatio

The mortality rate in the vaccinated group was significantly less than the control groups (*P*<0.05). In the groups immunized with *pEGFP-N1*-KMP11-*GP96* and pEGFP-N1- KMP11 the survival rates were higher compared to control groups (*P*< 0.05) (Fig. 8).

Only 20% of the mice that received pEGFP-N1-KMP11-*GP96* or *pEGFP-N1*- KMP11 dead during 30 wk after challenge where 100% of control groups dead within 27 wk. After 30 wk, no parasite was isolated from liver, bone marrow and spleen of the live mice. No side effect was observed after using the fusion in the live mice.

### Lesion size in immunized and control groups mice

Lesions with a mean size of 1.5 mm were observed at three weeks after challenge in the control groups; while, the mean size of observed lesions in the immunized group with pEGFP-N1-KMP11-*GP96* was 0.50 mm three weeks after the challenge. The mean size of the lesions in both vaccinated group was meaningfully smaller than those in the control group (*P*< 0.05) (Table 1). In mice vaccinated with pEGFP-N1-KMP11-*GP96* and pEGFPN1-KMP11, the cutaneous lesions were cured 197 and 201 d after challenge, respectively, while no healing was observed in the mice that received pEGFP-N1 and PBS.

**Table 1:** The lesion size of immunized BALB/c in different groups after challenge with 2×10^6^promastigotes of *L. major*

***Groups***	***Lesion size (mm)***	***Lesion size (mm)***	***Time of lesion healing (Day)***
pEGFP-KMP-GP96	Maximun	Mean ± SE	201
pEGFP-KMP	0.70	0.50± 0.22	197
pEGFP-N1	1.10	0.80± 0.25	0
PBS	20.15	17.60± 2.08	0

## Discussion

Drugs currently used to treat Leishmaniasis have a number of problems, including high toxicity and various side effects (21). Therefore, attempts to introduce new candidates for the production of effective drugs and vaccines are still ongoing.

In general, *Leishmania* vaccines are divided into 3 categories (22). The first-generation vaccines consist of dead parasites, which have gradually replaced leishmanization. The first-generation vaccine have been made using complete killed parasites or their extracts. Numerous efforts have been made to develop such vaccines in Brazil, Colombia, Ecuador, Venezuela and the Islamic Republic of Iran against leishmaniasis and in Sudan against visceral leishmaniasis .

Second-generation vaccines include genetically modified residual leishmaniasis, or bacteria or viruses carrying *Leishmania* antigen genes, or sub-units of synthetic or recombinant species, and native fractions purified from the parasite. Various proteins of *Leishmania* have been identified as antigens, *gp63* (2), *Hsp70* (3), *TSA* (4), *PSA*-2 (5), *Histone H1* (6), *LACK* (7, 8). Many of these antigens have been identified that, when combined with adjuvants, they are able to provide protection in experimental animals, but to date, only one vaccine has been obtained in clinical evaluations (23). The rest have been provided little immunity against more than one species in animal models.

Third-generation vaccines include genes encoding an immunogenic antigen cloned in a eukaryotic promoter vector. Compared to the recombinant vaccine, DNA vaccines are more stable and have advantages such as cheap production, lack of need for a cold chain and flexibility in combining different genes of different species. These vaccines induce both humoral and cellular immune responses, and these responses can be shifted by altering the vector or attaching cytokine genes with adjuvant properties. Therefore, the immune response to the encoded antigen from the plasmid can be located in a pathway that produces resistance to the parasite (24). These vaccines provide a better immunity against *Leishmania* compared to dead or live vaccines, as they can induce expression of different *Leishmania* antigens without any kind of alteration in structure and antigenicity. DNA vaccines are naturally immunogenetic due to the presence of non-methylated CpG patterns in their structure, leading to the expression of Th1 cytokines and increased TCD8 + cell responses (25). In recent years, antigens such as *LACK*, *LeIF*, *TSA*, *LmSTI1*, *H1*, *CPA* + C*P*B, *KMP-*11, and *NH36* have been used for production of third-generation vaccine against leishmaniasis.

Studies on *KMP*-11 protein have shown that *KMP*-11 clearly has three major immunological features: stimulation of B cell, inducing lymphocytic proliferation and cytotoxic response, and producing protective immunity in animal models. A study showed that immunoglobulin subtypes in response to KMP-11 protein include IgG1, IgG3, IgG2 and IgG4 (26). Some researchers have shown the ability of KMP-11 protein to induce proliferation of T-lymphocytes. KMP-11 of *L.donovani*, *T. rhodesiense*, *T. brucei*, *T. congolense* and *T. simiae* was a potent stimulator of CD4 +, CD8- in mice immunized with KMP-11 protein (16). Immunization of BALB/c mice with an attenuated strain of *Toxoplasma gondii*, which expresses the *Leishmania* KMP-11 protein, provides a specific and protective immune response in such animals (27). The evidence strongly suggests that this gene is an excellent target for immunotherapy and immunization against leishmaniasis.

*GP96* as one of the most abundant intracellular heat shock proteins possesses multiple functions and its ability to bridge the innate and adaptive immune systems has attracted extensive interest. The immunogenicity of *GP96* is dependent on its ability to bind peptide epitopes. Both the N- and C-terminal fragments of *GP96* are able to bind peptides, with the N-terminal fragment behaving at a similar capacity to the full-length *GP96* (20).

In our study, the constructed fusion showed a potency to elicit both Th1 &Th2 responses, but it showed the higher ratio of IFN- γ / IL-4 and IgG2a/IgG1 compare to control groups, indicating that the constructed fusion could well stimulate the cellular immune system in mice.

However, the role of Th1 was greater in the immune response against the infection. These results are in accordance with those of other reports that a higher ratio of the IgG2a/IgG1 isotype is associated with protective immunity against *L. major* infection in Balb/c mice. Nevertheless, of whether the healing resulted from low doses of parasites (28) or the healing resulted from immunization (29, 30).

## Conclusion

We successfully cloned fusion of two genes in pEGFP-N1 to use it as a DNA vaccine. The constructed fusion could well stimulate both the cellular and humoral immune systems that led to cutaneous lesion healing in mice.
